# Structural Insights into the Respiratory Syncytial Virus RNA Synthesis Complexes

**DOI:** 10.3390/v13050834

**Published:** 2021-05-05

**Authors:** Dongdong Cao, Yunrong Gao, Bo Liang

**Affiliations:** Department of Biochemistry, Emory University School of Medicine, Atlanta, GA 30322, USA; dongdong.cao@emory.edu (D.C.); yunrong.gao@emory.edu (Y.G.)

**Keywords:** structure, respiratory syncytial virus, RNA synthesis, gene transcription, genome replication, N, L, P, and M2-1

## Abstract

RNA synthesis in respiratory syncytial virus (RSV), a negative-sense (−) nonsegmented RNA virus, consists of viral gene transcription and genome replication. Gene transcription includes the positive-sense (+) viral mRNA synthesis, 5′-RNA capping and methylation, and 3′ end polyadenylation. Genome replication includes (+) RNA antigenome and (−) RNA genome synthesis. RSV executes the viral RNA synthesis using an RNA synthesis ribonucleoprotein (RNP) complex, comprising four proteins, the nucleoprotein (N), the large protein (L), the phosphoprotein (P), and the M2-1 protein. We provide an overview of the RSV RNA synthesis and the structural insights into the RSV gene transcription and genome replication process. We propose a model of how the essential four proteins coordinate their activities in different RNA synthesis processes.

## 1. Overview of the Respiratory Syncytial Virus (RSV)

The human respiratory syncytial virus (HRSV or RSV) is an enveloped virus with a negative-sense (−) genome in the genus *Orthopneumovirus*, family *Pneumoviridae*, order *Mononegavirales* [1]. Since its first isolation in 1955, RSV has been a leading cause of infant mortality by viral infections in the US and worldwide [2]. RSV is the most common cause of hospitalization of infants for respiratory problems, with an estimation of 80,000 cases each year in the US [3]. The RSV reinfection remains common throughout all age groups, causing bronchiolitis in infants, common colds in adults, and more serious respiratory illnesses in older adults and immunocompromised patients [4]. Unfortunately, no effective vaccine or antiviral therapy is available to prevent or treat RSV [5,6,7,8].

The order of *Mononegavirales* is also called nonsegmented negative-sense (NNS) RNA viruses, a class of pathogenic and sometimes deadly viruses that include measles (MeV), rabies (RABV), Nipah (NiV), Hendra (HeV), Ebola (EBOV), Marburg (MARV), and RSV. The family *Pneumoviridae* has two genera *Orthopneumovirus* and *Metapneumovirus*. The genus *Orthopneumovirus* consists of bovine RSV (BRSV), murine pneumonia virus (MPV), and human RSV. HRSV has two antigenic subgroups, A and B, with genome-wide sequence divergence. The genus *Metapneumovirus* consists of human metapneumovirus (HMPV) and avian metapneumovirus (AMPV) [1].

## 2. RSV Virion and Genome

The RSV virions are either spherical particles of 100–350 nm in diameter or long filaments up to 10 μm and 60–200 nm in diameter. The RSV virion comprises an RNA synthesis ribonucleoproteins (RNP) complex packaged in a lipid envelope derived from the host cell membrane. The RNA synthesis RNP consists of four proteins essential for the RSV RNA synthesis: the nucleoprotein (N), the large polymerase protein (L), the phosphoprotein (P), and the processivity factor M2-1. The RSV envelope contains three membrane proteins: the glycoprotein (G), the fusion protein (F), and the small hydrophobic protein (SH). The matrix protein (M) lays between the RNP and the envelope, acting as the cushion.

**Figure 1:**
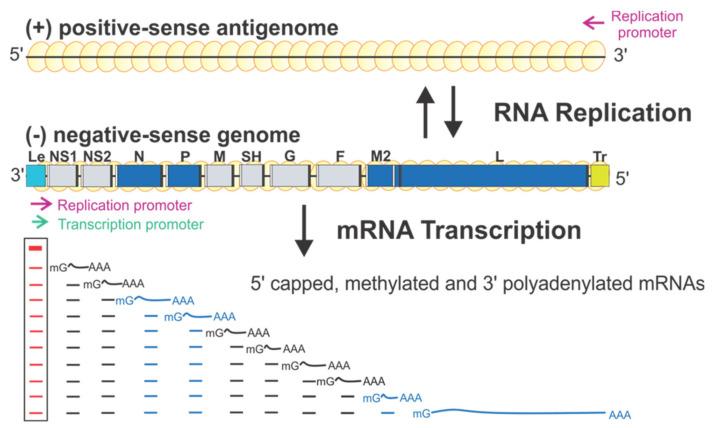
The schematic diagram of the genome organization and RNA synthesis of RSV. The leader (Le, cyan) and trailer (Tr, gold) are at 3′ and 5′ ends of the genome. The genes N, P, M2, L (blue), NS1, NS2, M, SH, G, and F (gray) are flanked with gene start (GS, white) and gene end (GE, black). Two replication promoters (magenta arrows) at 3′ ends of the genome and antigenome and a single transcription promoter (green arrow) are shown. The mRNA transcription products with 5′ methyl guanosine (^m^G) caps and 3′ poly-A (A_n_) are below respective genes. Genome and antigenome are encapsidated with N proteins (yellow circles).

RSV encodes ten sequential viral genes in the order of 3′ NS1–NS2–N–P–M–SH–G–F–M2–L, and each gene is flanked by conserved gene start (GS) and gene end (GE) sequences. The genes responsible for the RSV RNA synthesis are highlighted in blue (Figure 1). Each gene encodes an mRNA with the 5′ methylated cap and 3′ polyA tail to be translated into a single corresponding protein, except the M2 gene, which has two slightly overlapped open reading frames (ORFs) encoding two proteins: M2-1 and M2-2. Two extragenic regions are at the genome ends, a 3′ 44-nt leader (Le) and a 5′ 155-nt trailer (Tr).

## 3. RSV RNA Synthesis

**Figure 2:**
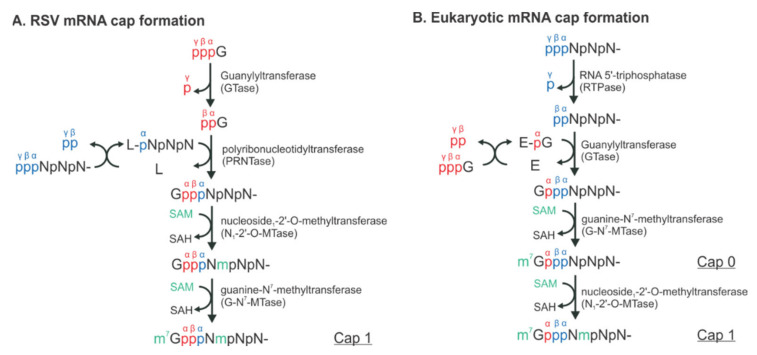
The distinct mechanisms of the RSV and eukaryotic mRNA cap formation. The schematical comparison of the unconventional (**A**) and conventional (**B**) pathways. The pre-mRNA, GTP, and S-adenosyl-L-methionine (SAM) are highlighted in blue, red, and green, respectively. The α, β, and γ positions of the phosphates are indicated to illustrate the exact positions where the chemical reactions occur. N denotes any of the four nucleotides, A, U, G, and C. L denotes the large protein L in RSV. E denotes the enzyme, guanylyltransferase (GTase). Note that there is an L:RNA (L-pNpNpN) intermediate and the final mRNA cap products of both unconventional and conventional pathways are chemically identical.

RSV initiates viral infection by delivering into the host cell a virus-specific RNA synthesis RNP required for both replications of the full-length genome and transcriptions of individual viral genes [9]. RNA synthesis is carried out by the RNA-dependent RNA polymerase (RdRp) complex, which consists of the catalytic core L and the cofactor P. L is a 250 kDa polypeptide that executes the synthesis of viral genomic or antigenomic RNAs and mRNAs and catalyzes three distinct enzymatic activities: ribonucleotide polymerization, mRNA 5′ cap addition, and cap methylation [6]. Interestingly, the mRNA caps are synthesized by unique chemical reactions: (a) the cap forms via a covalent L:RNA intermediate, distinct from eukaryotes and all other virus orders; (b) the cap is methylated at the 2′-O position first, followed by the N-7 position, the opposite order of mammalian mRNAs [10,11] (highlight in Figure 2). The RNA template for the polymerase is not naked RNA but rather a helical nucleocapsid (NC, N:RNA), a single-strand RNA continuously encapsidated by the N protein, as highlighted as yellow circles on (−) genome and positive-sense (+) antigenome (Figure 1). Each RSV N coats seven nucleotides (nts) [12,13], and the entire RSV genome of 15,222 nts (A2 strain, GenBank accession number M74568) requires more than 2000 copies of N.

There is only one promoter (green arrow) for transcription at the 3′ end of the Le region (position 1–11) (Figure 1). The polymerase initiates RNA synthesis at this promoter to produce an uncapped leader complementary (LeC) RNA and progresses along the genome. After recognizing the first GS signal, the polymerase initiates mRNA synthesis, caps and methylates the 5′ of the mRNA, and elongates it. When the polymerase reaches the GE signal, it polyadenylates the transcript using a short U-rich template, releases the nascent mRNA, and then reinitiates downstream mRNA synthesis at the next GS signal. There are two replication promoters (magenta arrows), one embedded in the Le sequence and another residing in the 3′ end of the (+) antigenome (Figure 1). During replication, the polymerase binds to the promoter in the Le region (position 1–34) and initiates RNA synthesis at the 3′ end. It copies the template to generate a full-length (+) RNA antigenome, ignoring the cis-acting GS and GE signals. At the 3′ end of the antigenome is the complement sequence of the trailer (TrC), which shares an 88% sequence identity with the Le and contains a promoter. The polymerase uses this promoter to generate (−) RNA genome progeny. The promoters for transcription and replication overlap but are different in length, and all promoters can be recognized by the same polymerase [12,14] (Figure 1).

Much of our current understanding of RNA synthesis comes from studies of prokaryotic or eukaryotic DNA- or RNA-dependent RNA polymerases [15,16,17,18]. RNA synthesis by RSV and other NNS RNA viruses is believed to follow the “two-metal-ion” mechanism of catalysis [19] but differs from the cellular polymerases for three main aspects. First, rather than naked RNA, the authentic RNA template is wrapped by N as a helical NC. Second, the N:RNA genome serves as the template for both transcription and replication, and the promoters of these two processes overlap, requiring the polymerase to be highly regulated. Third, instead of having a promoter for each gene, all viral mRNAs are synthesized from a single promoter, with the polymerase stopping and reinitiating synthesis along the genome. The RSV transcription and replication appear straightforward, requiring the RNA polymerase, the promoter at the 3′ end of the genome and antigenome RNAs, and short cis-acting signals flanking each of the genes.

Further, studies using the RSV minigenome replicon in which the RNP complexes are reconstituted in cells by supplying the trans-acting protein and RNA components have shown that the replication requires N, L, and P, whereas transcription requires N, L, P, and M2-1 [5,6]. Remarkably, using these minimal elements, RSV coordinates multiple biosynthetic events to generate appropriate ratios of encapsidated antigenome and genome RNAs, as well as multiple monocistronic 5′ capped, methylated, and 3′ polyadenylated viral mRNAs to ensure faithful virus propagation [9,20] (Figure 1). Reviews of RSV and NNS RNA viruses can be found in [5,6,9].

## 4. Structural Insights of the RSV RNA Synthesis Complex

In the past two decades, the advance of structural and biochemical studies on the *Mononegavirales* RNA synthesis RNPs has provided rich insights into RSV RNA synthesis and viral replication. For the comparison purpose, we use the well-studied vesicular stomatitis virus (VSV) and human parainfluenza virus (HPIV), the representative members of the *Rhabdoviridae* and *Paramyxoviridae* familes, respectively, and HMPV as a control in the *Pneumoviridae* family. Comparing the RNP structures of RSV and other representative viruses sheds light on the shared and different transcription and replication strategies. As highlighted above, N, L, P, and M2-1 are the minimal components for the *in vitro* reconstitution of the RSV RNA synthesis machinery. This review focuses on RSV and summarizes structural insights of those four proteins that constituted the RSV RNA synthesis RNP. Readers who are interested in more targeted reviews may read recommendations in each subsection.

### 4.1. The RSV L Protein

The RSV L is a single polypeptide of 2165 residues and is the catalytic core of the RSV polymerase. The RSV L exists as a monomeric but multifunctional enzyme, bearing three distinct enzymatic domains, namely, the RNA-dependent RNA polymerization (RdRp), the cap addition (Cap), and the cap methylation (MT) domains. Besides three functional domains, the RSV L also contains two structural domains, the connector domain (CD) and the C-terminal domain (CTD). The RSV RdRp activities share similarities with the activities of other viral RNA-dependent RNA polymerases (RdRPs), but the Cap and MT activities are unique to the *Mononegavirales* and distinct to the capping in the host cells.

**Figure 3:**
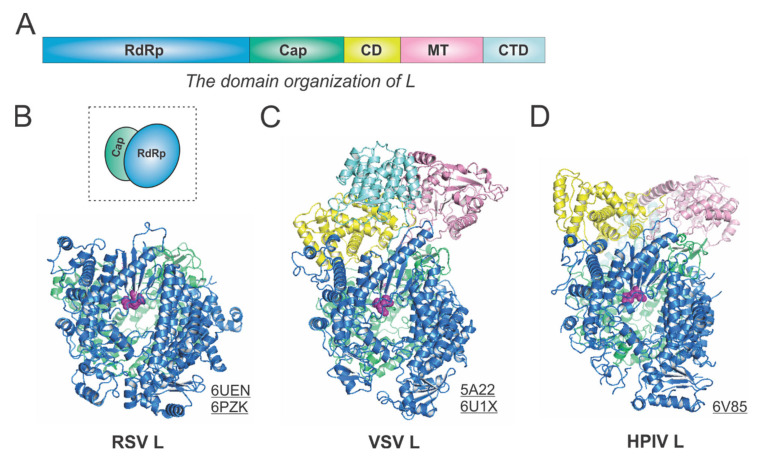
The structures of the L protein from RSV and other representative viruses. (**A**) The linear domain organization of the L protein. There are five domains, namely, the RNA-dependent RNA polymerization (RdRp, blue), the cap addition (Cap, green), the connector domain (CD, yellow), the cap methylation domain (MT, pink), and the C-terminal domain (CTD, cyan). (**B**) The model of the RSV L is extracted from the structures of the RSV polymerase (PDBs: 6UEN and 6PZK). The cartoon representation of the RSV L is shown in the dotted box. (**C**) The model of the VSV L is extracted from the structures of the VSV polymerase (PDBs: 5A22 and 6U1X). (**D**) The model of the HPIV L is extracted from the structure of the HPIV polymerase (PDB: 6V85). The PDB accession codes are underlined.

The RdRp, Cap, CD, MT, and CTD domain organizations of *Mononegavirales* L are color-coded in blue, green, yellow, pink, and cyan, respectively, in Figure 3A. For the determined structures of the RSV polymerase, only the RdRp and Cap domains of the RSV L are visible, with the catalytic sites of the RdRp domains highlighted in magenta spheres [21,22] (Figure 3B). The cartoon representation of the RSV L is shown in the dotted box. Consistent with this, the closely related HMPV polymerase structure also lacks the CD, MT, and CTD domains for L [23]. In contrast, both VSV L and HPIV L structures contain all five domains [24,25,26] (Figure 3C,D). Note that all three structures presented here are superposed based on the RdRp domain. Interestingly, there is a domain swap of the MT and CTD domains for the VSV L and HPIV L [26,27]. Readers interested in prior reviews on viral and *Mononegavirales* RdRPs may consult [27,28,29], and the unique capping mechanisms can be found in [10,11].

### 4.2. The RSV P Protein

**Figure 4:**
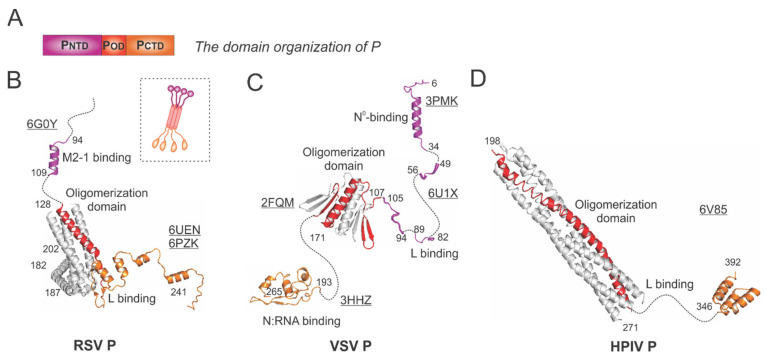
The structures of the phosphoprotein (P) from RSV and other representative viruses. (**A**) The linear domain organization of the P protein. There are three domains: the N-terminal domain P_NTD_ (magenta), the oligomerization domain P_OD_ (red), and the C-terminal domain P_CTD_ (orange). (**B**) The models of the RSV P are extracted from the structures of the RSV polymerase (PDBs: 6UEN and 6PZK) and the P:M2-1 complex (PDB: 6G0Y). The cartoon representation of the RSV P is shown in the dotted box. (**C**) The models of the VSV P are extracted from the structures of the VSV polymerase (PDB: 6U1X), P_OD_ (PDB: 2FQM), N°-binding domain at P_NTD_ (PDB: 3PMK), and N:RNA-binding domain at P_CTD_ (PDB: 3HHZ). (**D**) The model of the HPIV P is extracted from the structure of the HPIV polymerase (PDB: 6V85). Note: The terminal residue numbers are indicated, and the flexible or unmodeled regions are linked by the dotted lines. Only one protomer is colored as panel A, and other protomers are colored in gray for clarity. The PDB accession codes are underlined.

In general, the P protein contains three domains: the N-terminal domain (NTD), the oligomerization domain (OD), and the C-terminal domain (CTD), which are indicated as P_NTD_ (magenta), P_OD_ (red), and P_CTD_ (orange), respectively (Figure 4A).

The RSV P is a protein of 241 residues with multiple flexible regions and the essential cofactor of the RSV polymerase. The RSV P exists as a tetrameric protein, acting as the multimodular adapter to coordinate the RNA synthesis complex activities and interacting with RNA-free N (N°), N:RNA complex, and M2-1 proteins [5,6]. Thus far, the structures of the RSV polymerase (L:P) and the RSV M2-1:P complex have been determined [21,22,30]. The cartoon representation of the RSV P is shown in the dotted box (Figure 4B). The ribbon diagrams of P_NTD_ that interacts with M2-1 and P_OD_ (red) and P_CTD_ that binds to L are shown with the terminal residue numbers indicated. The flexible or unmodeled regions are linked by the dotted lines, and only one copy of the M2-1-binding domain is shown (Figure 4B). The M2-1-binding motif of P on M2-1 is illustrated in Section 4.4.

**Figure 5:**
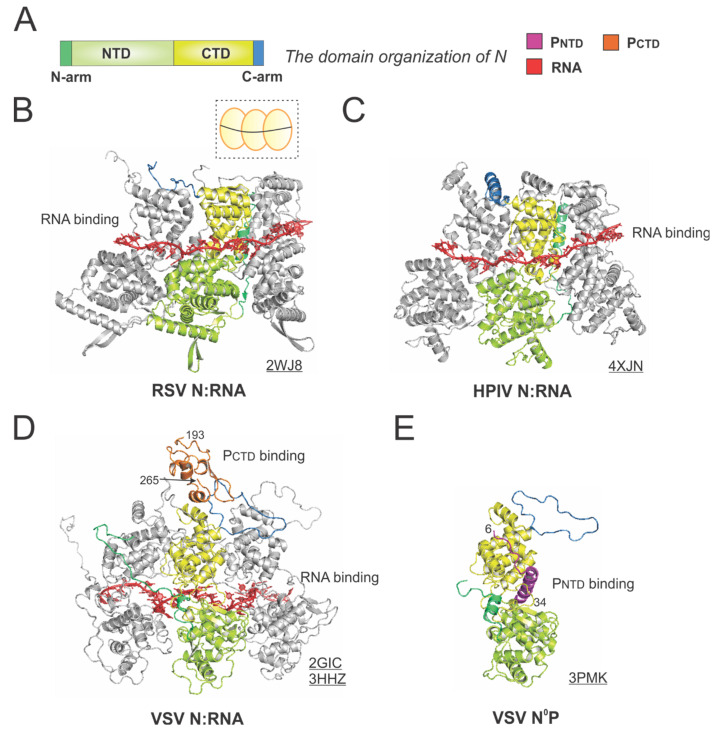
The structures of the nucleoprotein (N) or nucleocapsid (NC) from RSV and other representative viruses. (**A**) The linear domain organization of the N protein. There are two core domains: the N-terminal domain (NTD, light green) and the C-terminal domain (CTD, yellow), plus the N-terminal motif (N-arm, green) and C-terminal motif (C-arm, blue) near the terminus of the core domains. (**B**) The model of the RSV N:RNA is extracted from the crystal structure of the RSV N:RNA (PDB: 2WJ8). The cartoon representation of the RSV N:RNA is shown in the dotted box, with N as the yellow circles (consistent with Figure 1) and the RNA as the black line. (**C**) The model of the HPIV N:RNA is extracted from the crystal structure of the HPIV N:RNA (PDB: 4XJN). (**D**) The models of the VSV N:RNA are extracted from the crystal structure of the VSV N:RNA and NC:P (PDBs: 2GIC, 3HHZ). The P_CTD_ is colored in orange, consistent with Figure 4. (**E**) The crystal structure of the VSV N°P (PDB: 3PMK). Three protomers are shown in the N:RNA structures, and only the middle protomer is colored as the domain organization, and the rest are in gray. The PDB accession codes are underlined.

The VSV P is the most extensively studied among *Mononegavirales* P proteins. Previously, the co-crystal structures of the N°-binding motif, the N:RNA-binding motif, and the oligomerization domain of P interacting with respective binding partners have been determined [31,32,33] (Figure 4C). Recently, the cryo-EM structure of the VSV polymerase revealed several fragments of P_NTD_ that interact with the CTD, RdRp, and CD domains of L [24]. Again, the flexible or unmodeled regions are linked by the dotted line, with the terminal residue numbers indicated (Figure 4C). The architectures of the VSV NC:P and N°P complexes highlight the N°-binding and the N:RNA-binding motif, respectively (Figure 5D,E).

The HPIV P model was extracted from the structure of the HPIV polymerase [26] (Figure 4D). P_OD_ (red) sits on the RdRp domain of HPIV L (Figure 3D) and points away from it, and the P_CTD_ (orange) of one chain of P also interacts with L. Interestingly, there are several notable differences among the P proteins presented here from different families: (1) the RSV P and HPIV P are tetramers, but the VSV P is a dimer; (2) the length of the oligomerization domain of HPIV P is more than twice the length of the RSV P_OD_ or VSV P_OD_; (3) the sizes of the RSV P and VSV P are similar (241 aa vs. 265 aa), but HPIV P is much longer (392 aa).

Due to the flexibility of the P_NTD_ and P_CTD_, only structures of P_OD_ were readily available from other *Mononegavirales*, including those of RABV, HMPV, MeV, MuV, SeV, NiV, and MARV [34,35,36,37,38,39,40] (See the structural comparison in [21]).

### 4.3. The RSV N Protein

The RSV N is a protein of 391 residues, consisting of two core domains: the N-terminal domain (NTD, light green) and the C-terminal domain (CTD, yellow), plus the N-terminal motif (N-arm, green) and C-terminal motif (C-arm, blue) near the terminus of the core domains (Figure 5A).

The crystal structure of the RSV N:RNA pseudo-ring revealed that N has two core lobes (NTD and CTD) with the RNA bound in the central groove, and each N coats 7-nt of RNA [13]. Three continuous N subunits are shown, with the middle N highlighted in color and the left and right N molecules in gray. The interacting RNA molecules are colored in red (Figure 5B). Both the N-arm and C-arm of N connect the adjacent N subunits in the RNA-bound ring, providing a significant stabilizing interaction. The cartoon representation of the RSV N:RNA is shown in the dotted box (Figure 5B). The structure of HPIV N:RNA is also shown with a similar orientation. The N-arm and C-arm of the HPIV N are slightly different from that of the RSV N, but the location and the role are similar [41] (Figure 5C).

The first high-resolution structures of the *Mononegavirales* N:RNA were the VSV N:RNA by Green et al. and RABV viral N:RNA by Albertini et al. in 2006 [42,43], providing the first glimpses of how RNA is encapsidated by N (Figure 5D). Both VSV and rabies belong to the family Rhabdoviridae. Their structures are similar, with every N protein coating nine nucleotides, although the VSV N:RNA is a 10-mer ring, while RABV N:RNA is an 11-mer ring. After that, several other *Mononegavirales* N:RNA structures were determined, including those of RSV, HMPV, HPIV, MeV, and EBOV [13,41,44,45,46,47]. The Luo group also determined the structure of P_CTD_ that binds to the VSV N:RNA [32]. The structure revealed that the P_CTD_ (orange) binds to the CTD and the C-arm of two adjacent N proteins (Figure 5D). Interestingly, the study on the interaction between the RSV P_CTD_ to the RSV N suggests that in RSV, P_CTD_ interacts with N_NTD_, rather than N_CTD_, which may be unique for *Pneumoviridae* N proteins [48,49].

Biochemical studies suggest P binds to RNA-free N (N°) monomers and delivers them to nascent RSV genomes or antigenomes [50]. The P_NTD_ inhibits N self-assembly and acts as a chaperone for monomeric N. In 2011, Leyrat et al. determined the co-crystal structure of the VSV N°P [31]. While the N is in the same orientation as in the N:RNA structure, the P_NTD_ (magenta) binds to a similar location where the RNA is located in the N:RNA complex (Figure 5E). The structures of the N°P from other *Mononegavirales*, such as HMPV, HPIV, MeV, NiV, have also been determined, providing additional insights on such interactions [44,51,52,53]. Briefly, the available evidence highlights the shared role of the P_NTD_ in interacting with N°. Utilizing this feature, recently, in our group and in others, the P_NTD_ is co-expressed with N to generate N°, which can be used for *de novo* assembly of virus-specific RNA templates [54,55,56]. Readers interested in prior reviews on the N and nucleocapsid structures of *Mononegavirales* may consult [57,58,59].

### 4.4. The RSV M2-1 Protein

M2-1 acts as an antiterminator to ensure the full-length transcription of all ten RSV mRNAs, and it is impossible to rescue infectious RSV from cDNAs without M2-1 [60]. M2-1 exists as a tetramer in solution, and the early study showed M2-1 directly interacts with RNA and P in a competitive manner [61]. However, it is not clear whether the interaction with RNA and P is strictly exclusive.

**Figure 6:**
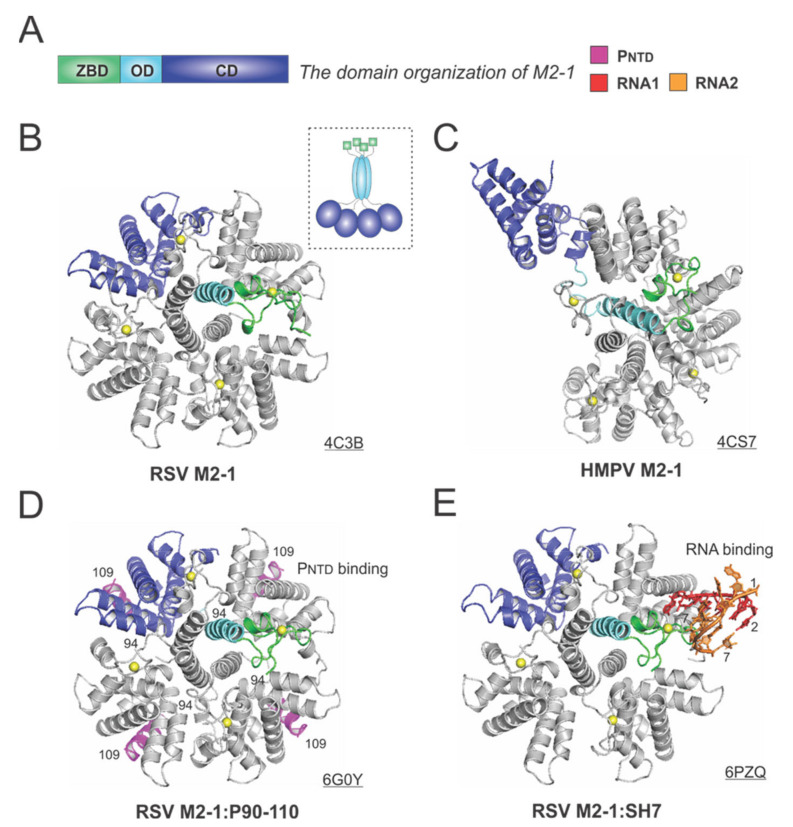
The structures of the M2-1 protein from RSV and HMPV. (**A**) The linear domain organization of the M2-1 protein. There are three domains: the zinc-binding domain (ZBD, green), an oligomerization domain (OD, cyan), and a core domain (CD, blue). (**B**) The crystal structure of the RSV M2-1 (PDB: 4C3B). The cartoon representation of the RSV M2-1 is shown in the dotted box. (**C**) The crystal structure of the HMPV M2-1 (PDB: 4CS7). Note that the protomer in the open state is shown as the same color scheme as in panel A. (**D**) The crystal structure of the RSV M2-1 in the complex of the P_NTD_ (PDB: 6G0Y). The P_NTD_ is highlighted in magenta, consistent with Figure 4. (**E**) The crystal structure of the RSV M2-1 in the complex of a short RNA oligo (PDB: 6PZQ). Two RNA molecules are colored in red and orange, respectively. Four protomers are shown in the M2-1 structures. Only one protomer is colored as the domain organization, and the rest are in gray. The PDB accession codes are underlined.

The 194-residue RSV M2-1 consists of three distinct domains: the zinc-binding domain (ZBD: 1–31), an oligomerization domain (OD: 32–68), and a core domain (CD: 69–194). The domain organization of M2-1 is shown in Figure 6A. In 2014, Tanner et al. determined the crystal structures of *apo* RSV M2-1 in two different space groups, which revealed the symmetrical tetramer configuration [62] (Figure 6B). The cartoon representation of the RSV M2-1 is shown in the dotted box. Interestingly, Leyrat et al. determined that the crystal structure of a related *apo* HMPV M2-1 showed an asymmetric tetramer, with three of the protomers in a closed conformation and one protomer in an open conformation [63] (Figure 6C). The crystal structures of the HMPV M2-1 in complex with adenosine monophosphate (AMP) and 5-nt DNA fragments were also determined, revealing that the RNA surface binding sites are consistent with the NMR and mutagenesis studies [63]. Readers interested in a prior review on RSV M2-1 may consult [64].

Two recent studies revealed more details on the interactions between M2-1 and P or RNA. In 2018, Selvaraj et al. determined the co-crystal structure of M2-1:P(90–110), showing the location of P(90–110) fragment on the surface of M2-1 [30] (Figure 6D). In the same study, it was demonstrated that high-affinity RNAs could outcompete P [30]. More recently, in 2020, our group determined a co-crystal structure of M2-1 bound to a short positive-sense gene-end RNA (SH7) [65]. We used both experiments and simulations to reveal that RNA interacts with two separate domains, ZBD and CD, of M2-1, independent of each other [65] (Figure 6E). It was shown that M directly interacts with M2-1, and M2-1 is likely to form a layer below M while interacting with the RNP complex [66]. Recent fluorescence microscopy studies by Bouillier et al. have shown M2-1 is colocalized at the inclusion body-associated granule (IBAG), the site of active viral RNA synthesis [67].

## 5. The Model of RSV RNA Synthesis

**Figure 7:**
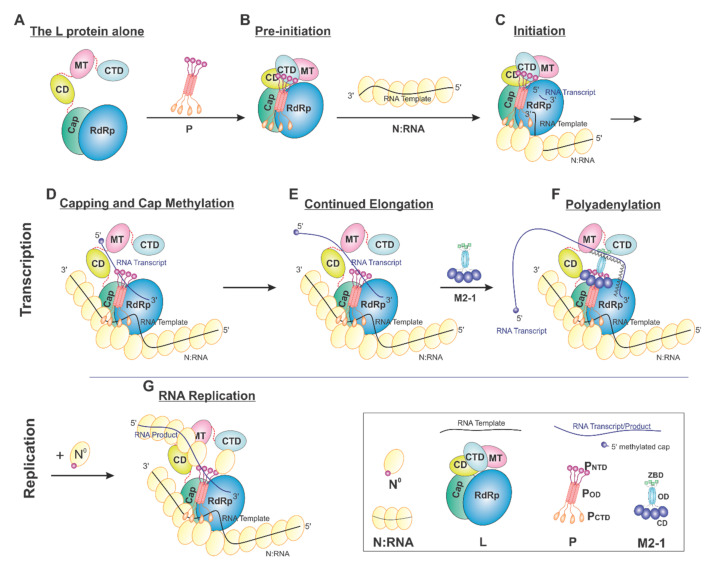
Proposed cartoon models of RSV RNA synthesis. The RNA template, RNA transcript, and the flexible linker are shown in the black, blue, and red lines, respectively. (**A**) The L protein alone: The cartoon depicting the conformational flexibility of the connector domain (CD), the methyltransferase domain (MT), and the C-terminal domain (CTD) of L. (**B**) Pre-initiation: conformational rearrangements of the flexible domains upon binding of the tetrameric P protein. (**C**) Initiation: the priming loop of the Cap domain is at the close approximate of the *de novo* initiation site of the RdRp domain. (**D**) Capping and cap methylation: the Cap and flexible domains, in particular, the MT domain, rearranged to catalyze the cap addition and cap methylation. (**E**) Continued elongation: after the proper cap, the elongation stage of the transcription continues. The flexible domains again become flexible. (**F**) Polyadenylation: the recruitment of M2-1 when polyA transcript is synthesized. (**G**) RNA replication: upon the supply of the N° (RNA-free N), the RNA replication continues.

On the basis of current data, we proposed a model of the sequential RSV RNA synthesis (Figure 7). In general, L alone adopts an open conformation (A). P coordinates the activity of L and shows a previously unappreciated role for L domain arrangements. Upon binding, a tetrameric P locks CD, MT, and CTD (gray) domains of L (pre-initiation) into a closed conformation (B). The pre-initiation complex then recruits the N:RNA to form the initiation complex and start the *de novo* RNA synthesis (C). These domains then adopt an open conformation upon promoter recognition and remain open during elongation (D–G), resulting in higher mobility of the CD, MT, and CTD domains of L. Depending on N°, RNA synthesis goes to either transcription (capping and methylation, continued elongation, and polyadenylation, D–F) or replication (G). Even within this proposed framework, some ambiguity remains. For example, (1) P may bind to L all the time. (2) The domains of L undergo significant conformational rearrangements in response to not only P but also other cofactors, such as M2-1 and N. (3) There is also the possibility that the active RNA synthesis needs the coordination of multiple (e.g., two) L proteins at the same time.

## 6. Conclusions and Discussion

Undoubtedly, the visualization of the RSV RNA synthesis RNP has provided enriched insights into how RSV integrates viral transcription and replication as the essential part of the viral life cycle. The elucidation of the interaction surfaces and catalytic domains of the RNA synthesis RNP distinct from the host cell counterparts will facilitate the rational design of novel therapeutics [11,68,69,70]. The prior studies mainly focused on the atomic details of one or two components of the RNA synthesis RNP at a time. It is worthy to note that one highly dynamic field of the functional aspects of viral replication is the formation of membraneless liquid organelles, such as in the work demonstrated by Rincheval et al. [71]. Future studies will gear toward understanding how all four proteins are orchestrated in the assembly and function of the RSV RNA synthesis RNP at the molecular level. In particular, how the RSV RNA synthesis RNP (1) carries out the replication in a complex of the authentic N:RNA template, (2) executes the transcriptional steps in a step-wise manner, and (3) is regulated in the absences and presence of M2-1 is of great interest.

## Data Availability

Not applicable.

## References

[1] Maes P., Amarasinghe G.K., Ayllón M.A., Basler C.F., Bavari S., Blasdell K.R., Briese T., Brown P.A., Bukreyev A., Balkema-Buschmann A. (2019). Taxonomy of the order Mononegavirales: Second update 2018. Arch. Virol..

[2] Shi T., McAllister D.A., O’Brien K.L., Simoes E.A.F., Madhi S.A., Gessner B.D., Polack F.P., Balsells E., Acacio S., Aguayo C. (2017). Global, regional, and national disease burden estimates of acute lower respiratory infections due to respiratory syncytial virus in young children in 2015: A systematic review and modelling study. Lancet.

[3] McLaughlin J.M., Khan F., Schmitt H.J., Agosti Y., Jodar L., Simoes E.A.F., Swerdlow D.L. (2020). Respiratory Syncytial Virus-Associated Hospitalization Rates among US Infants: A Systematic Review and Meta-Analysis. J. Infect. Dis..

[4] Coultas J.A., Smyth R., Openshaw P.J. (2019). Respiratory syncytial virus (RSV): A scourge from infancy to old age. Thorax.

[5] Collins P.L., Karron R.A. (2013). Respiratory syncytial virus and metapneumovirus. Fields Virology.

[6] Collins P.L., Fearns R., Graham B.S. (2013). Respiratory Syncytial Virus: Virology, Reverse Genetics, and Pathogenesis of Disease. Curr. Top. Microbiol. Immunol..

[7] Jorquera P.A., Tripp R.A. (2017). Respiratory syncytial virus: Prospects for new and emerging therapeutics. Expert Rev. Respir. Med..

[8] Griffiths C., Drews S.J., Marchant D.J. (2017). Respiratory Syncytial Virus: Infection, Detection, and New Options for Prevention and Treatment. Clin. Microbiol. Rev..

[9] Whelan S.P.J., Barr J.N., Wertz G.W. (2004). Transcription and Replication of Nonsegmented Negative-Strand RNA Viruses. Curr. Top. Microbiol. Immunol..

[10] Ogino T., Banerjee A.K. (2007). Unconventional Mechanism of mRNA Capping by the RNA-Dependent RNA Polymerase of Vesicular Stomatitis Virus. Mol. Cell.

[11] Ogino T., Green T.J. (2019). RNA Synthesis and Capping by Non-segmented Negative Strand RNA Viral Polymerases: Lessons from a Prototypic Virus. Front. Microbiol..

[12] Cowton V.M., McGivern D.R., Fearns R. (2006). Unravelling the complexities of respiratory syncytial virus RNA synthesis. J. Gen. Virol..

[13] Tawar R.G., Duquerroy S., Vonrhein C., Varela P.F., Damier-Piolle L., Castagné N., MacLellan K., Bedouelle H., Bricogne G., Bhella D. (2009). Crystal Structure of a Nucleocapsid-Like Nucleoprotein-RNA Complex of Respiratory Syncytial Virus. Science.

[14] Noton S.L., Fearns R. (2015). Initiation and regulation of paramyxovirus transcription and replication. Virology.

[15] Kornberg R.D. (2007). The molecular basis of eukaryotic transcription. Proc. Natl. Acad. Sci. USA.

[16] Sekine S.-I., Tagami S., Yokoyama S. (2012). Structural basis of transcription by bacterial and eukaryotic RNA polymerases. Curr. Opin. Struct. Biol..

[17] Eick D., Wedel A., Heumann H. (1994). From initiation to elongation: Comparison of transcription by prokaryotic and eukaryotic RNA polymerases. Trends Genet..

[18] Cheetham G.M., Steitz T.A. (2000). Insights into transcription: Structure and function of single-subunit DNA-dependent RNA polymerases. Curr. Opin. Struct. Biol..

[19] Steitz T.A., Steitz J.A. (1993). A general two-metal-ion mechanism for catalytic RNA. Proc. Natl. Acad. Sci. USA.

[20] Conzelmann K.-K. (1998). Nonsegmented Negative-Strand RNA viruses: Genetics and Manipulation of Viral Genomes. Annu. Rev. Genet..

[21] Cao D., Gao Y., Roesler C., Rice S., D’Cunha P., Zhuang L., Slack J., Domke M., Antonova A., Romanelli S. (2020). Cryo-EM structure of the respiratory syncytial virus RNA polymerase. Nat. Commun..

[22] Gilman M.S., Liu C., Fung A., Behera I., Jordan P., Rigaux P., Ysebaert N., Tcherniuk S., Sourimant J., Eléouët J.-F. (2019). Structure of the Respiratory Syncytial Virus Polymerase Complex. Cell.

[23] Pan J., Qian X., Lattmann S., El Sahili A., Yeo T.H., Jia H., Cressey T., Ludeke B., Noton S., Kalocsay M. (2020). Structure of the human metapneumovirus polymerase phosphoprotein complex. Nat. Cell Biol..

[24] Jenni S., Bloyet L.-M., Diaz-Avalos R., Liang B., Whelan S.P., Grigorieff N., Harrison S.C. (2020). Structure of the Vesicular Stomatitis Virus L Protein in Complex with Its Phosphoprotein Cofactor. Cell Rep..

[25] Liang B., Li Z., Jenni S., Rahmeh A.A., Morin B.M., Grant T., Grigorieff N., Harrison S.C., Whelan S.P. (2015). Structure of the L Protein of Vesicular Stomatitis Virus from Electron Cryomicroscopy. Cell.

[26] Abdella R., Aggarwal M., Okura T., Lamb R.A., He Y. (2020). Structure of a paramyxovirus polymerase complex reveals a unique methyltransferase-CTD conformation. Proc. Natl. Acad. Sci. USA.

[27] Liang B. (2020). Structures of the Mononegavirales Polymerases. J. Virol..

[28] Ferrero D., Ferrer-Orta C., Verdaguer N. (2018). Viral RNA-Dependent RNA Polymerases: A Structural Overview. Subcell. Biochem..

[29] Peersen O.B. (2019). A Comprehensive Superposition of Viral Polymerase Structures. Viruses.

[30] Selvaraj M., Yegambaram K., Todd E., Richard C.A., Dods R.L., Pangratiou G.M., Trinh C.H., Moul S.L., Murphy J.C., Mankouri J. (2018). The Structure of the Human Respiratory Syncytial Virus M2-1 Protein Bound to the Interaction Domain of the Phosphoprotein P Defines the Orientation of the Complex. mBio.

[31] Leyrat C., Yabukarski F., Tarbouriech N., Ribeiro E.A., Jensen M.R., Blackledge M., Ruigrok R.W., Jamin M. (2011). Structure of the vesicular stomatitis virus N(0)-P complex. PLoS Pathog..

[32] Green T.J., Luo M. (2009). Structure of the vesicular stomatitis virus nucleocapsid in complex with the nucleocapsid-binding domain of the small polymerase cofactor, P. Proc. Natl. Acad. Sci. USA.

[33] Ding H., Green T.J., Lu S., Luo M. (2006). Crystal structure of the oligomerization domain of the phosphoprotein of vesicular stomatitis virus. J. Virol..

[34] Ivanov I., Crepin T., Jamin M., Ruigrok R.W. (2010). Structure of the dimerization domain of the rabies virus phosphoprotein. J. Virol..

[35] Leyrat C., Renner M., Harlos K., Grimes J.M. (2013). Solution and crystallographic structures of the central region of the phosphoprotein from human metapneumovirus. PLoS ONE.

[36] Communie G., Crepin T., Maurin D., Jensen M.R., Blackledge M., Ruigrok R.W. (2013). Structure of the tetramerization domain of measles virus phosphoprotein. J. Virol..

[37] Cox R., Green T.J., Purushotham S., Deivanayagam C., Bedwell G.J., Prevelige P.E., Luo M. (2013). Structural and functional characterization of the mumps virus phosphoprotein. J. Virol..

[38] Tarbouriech N., Curran J., Ruigrok R.W., Burmeister W.P. (2000). Tetrameric coiled coil domain of Sendai virus phosphoprotein. Nat. Struct. Biol..

[39] Bruhn J.F., Barnett K.C., Bibby J., Thomas J.M., Keegan R.M., Rigden D.J., Bornholdt Z.A., Saphire E.O. (2014). Crystal structure of the nipah virus phosphoprotein tetramerization domain. J. Virol..

[40] Bruhn J.F., Kirchdoerfer R.N., Urata S.M., Li S., Tickle I.J., Bricogne G., Saphire E.O. (2017). Crystal Structure of the Marburg Virus VP35 Oligomerization Domain. J. Virol..

[41] Alayyoubi M., Leser G.P., Kors C.A., Lamb R.A. (2015). Structure of the paramyxovirus parainfluenza virus 5 nucleoprotein-RNA complex. Proc. Natl. Acad. Sci. USA.

[42] Green T.J., Zhang X., Wertz G.W., Luo M. (2006). Structure of the vesicular stomatitis virus nucleoprotein-RNA complex. Science.

[43] Albertini A.A., Wernimont A.K., Muziol T., Ravelli R.B., Clapier C.R., Schoehn G., Weissenhorn W., Ruigrok R.W. (2006). Crystal structure of the rabies virus nucleoprotein-RNA complex. Science.

[44] Renner M., Bertinelli M., Leyrat C., Paesen G.C., Saraiva de Oliveira L.F., Huiskonen J.T., Grimes J.M. (2016). Nucleocapsid assembly in pneumoviruses is regulated by conformational switching of the N protein. Elife.

[45] Gutsche I., Desfosses A., Effantin G., Ling W.L., Haupt M., Ruigrok R.W., Sachse C., Schoehn G. (2015). Structural virology. Near-atomic cryo-EM structure of the helical measles virus nucleocapsid. Science.

[46] Sugita Y., Matsunami H., Kawaoka Y., Noda T., Wolf M. (2018). Cryo-EM structure of the Ebola virus nucleoprotein-RNA complex at 3.6 A resolution. Nature.

[47] Kirchdoerfer R.N., Saphire E.O., Ward A.B. (2019). Cryo-EM structure of the Ebola virus nucleoprotein-RNA complex. Acta Crystallogr. F Struct. Biol. Commun..

[48] Galloux M., Tarus B., Blazevic I., Fix J., Duquerroy S., Eleouet J.F. (2012). Characterization of a viral phosphoprotein binding site on the surface of the respiratory syncytial nucleoprotein. J. Virol..

[49] Ouizougun-Oubari M., Pereira N., Tarus B., Galloux M., Lassoued S., Fix J., Tortorici M.A., Hoos S., Baron B., England P. (2015). A Druggable Pocket at the Nucleocapsid/Phosphoprotein Interaction Site of Human Respiratory Syncytial Virus. J. Virol..

[50] Castagne N., Barbier A., Bernard J., Rezaei H., Huet J.C., Henry C., Costa B.D., Eleouet J.F. (2004). Biochemical characterization of the respiratory syncytial virus P-P and P-N protein complexes and localization of the P protein oligomerization domain. J. Gen. Virol..

[51] Aggarwal M., Leser G.P., Kors C.A., Lamb R.A. (2018). Structure of the Paramyxovirus Parainfluenza Virus 5 Nucleoprotein in Complex with an Amino-Terminal Peptide of the Phosphoprotein. J. Virol..

[52] Guryanov S.G., Liljeroos L., Kasaragod P., Kajander T., Butcher S.J. (2015). Crystal Structure of the Measles Virus Nucleoprotein Core in Complex with an N-Terminal Region of Phosphoprotein. J. Virol..

[53] Yabukarski F., Lawrence P., Tarbouriech N., Bourhis J.M., Delaforge E., Jensen M.R., Ruigrok R.W., Blackledge M., Volchkov V., Jamin M. (2014). Structure of Nipah virus unassembled nucleoprotein in complex with its viral chaperone. Nat. Struct. Mol. Biol..

[54] Milles S., Jensen M.R., Communie G., Maurin D., Schoehn G., Ruigrok R.W., Blackledge M. (2016). Self-Assembly of Measles Virus Nucleocapsid-like Particles: Kinetics and RNA Sequence Dependence. Angew Chem. Int. Ed. Engl..

[55] Desfosses A., Milles S., Jensen M.R., Guseva S., Colletier J.P., Maurin D., Schoehn G., Gutsche I., Ruigrok R.W.H., Blackledge M. (2019). Assembly and cryo-EM structures of RNA-specific measles virus nucleocapsids provide mechanistic insight into paramyxoviral replication. Proc. Natl. Acad. Sci. USA.

[56] Gao Y., Cao D., Ahn H.M., Swain A., Hill S., Ogilvie C., Kurien M., Rahmatullah T., Liang B. (2020). *In vitro* trackable assembly of RNA-specific nucleocapsids of the respiratory syncytial virus. J. Biol. Chem..

[57] Jamin M., Yabukarski F. (2017). Nonsegmented Negative-Sense RNA Viruses-Structural Data Bring New Insights into Nucleocapsid Assembly. Adv. Virus Res..

[58] Luo M., Terrell J.R., McManus S.A. (2020). Nucleocapsid Structure of Negative Strand RNA Virus. Viruses.

[59] Ruigrok R.W., Crepin T., Kolakofsky D. (2011). Nucleoproteins and nucleocapsids of negative-strand RNA viruses. Curr. Opin. Microbiol..

[60] Collins P.L., Hill M.G., Camargo E., Grosfeld H., Chanock R.M., Murphy B.R. (1995). Production of infectious human respiratory syncytial virus from cloned cDNA confirms an essential role for the transcription elongation factor from the 5′ proximal open reading frame of the M2 mRNA in gene expression and provides a capability for vaccine development. Proc. Natl. Acad. Sci. USA.

[61] Blondot M.L., Dubosclard V., Fix J., Lassoued S., Aumont-Nicaise M., Bontems F., Eleouet J.F., Sizun C. (2012). Structure and functional analysis of the RNA- and viral phosphoprotein-binding domain of respiratory syncytial virus M2-1 protein. PLoS Pathog..

[62] Tanner S.J., Ariza A., Richard C.A., Kyle H.F., Dods R.L., Blondot M.L., Wu W., Trincao J., Trinh C.H., Hiscox J.A. (2014). Crystal structure of the essential transcription antiterminator M2-1 protein of human respiratory syncytial virus and implications of its phosphorylation. Proc. Natl. Acad. Sci. USA.

[63] Leyrat C., Renner M., Harlos K., Huiskonen J.T., Grimes J.M. (2014). Drastic changes in conformational dynamics of the antiterminator M2-1 regulate transcription efficiency in Pneumovirinae. Elife.

[64] Muniyandi S., Pangratiou G., Edwards T.A., Barr J.N. (2018). Structure and Function of the Human Respiratory Syncytial Virus M2-1 Protein. Subcell Biochem..

[65] Gao Y., Cao D., Pawnikar S., John K.P., Ahn H.M., Hill S., Ha J.M., Parikh P., Ogilvie C., Swain A. (2020). Structure of the Human Respiratory Syncytial Virus M2-1 Protein in Complex with a Short Positive-Sense Gene-End RNA. Structure.

[66] Kiss G., Holl J.M., Williams G.M., Alonas E., Vanover D., Lifland A.W., Gudheti M., Guerrero-Ferreira R.C., Nair V., Yi H. (2014). Structural analysis of respiratory syncytial virus reveals the position of M2-1 between the matrix protein and the ribonucleoprotein complex. J. Virol..

[67] Bouillier C., Cosentino G., Leger T., Rincheval V., Richard C.A., Desquesnes A., Sitterlin D., Blouquit-Laye S., Eleouet J.F., Gault E. (2019). The Interactome analysis of the Respiratory Syncytial Virus protein M2-1 suggests a new role in viral mRNA metabolism post-transcription. Sci. Rep..

[68] Cox R., Plemper R.K. (2015). The paramyxovirus polymerase complex as a target for next-generation anti-paramyxovirus therapeutics. Front. Microbiol..

[69] Fearns R., Deval J. (2016). New antiviral approaches for respiratory syncytial virus and other mononegaviruses: Inhibiting the RNA polymerase. Antivir. Res..

[70] Cox R., Plemper R.K. (2016). Structure-guided design of small-molecule therapeutics against RSV disease. Expert Opin. Drug Discov..

[71] Rincheval V., Lelek M., Gault E., Bouillier C., Sitterlin D., Blouquit-Laye S., Galloux M., Zimmer C., Eleouet J.F., Rameix-Welti M.A. (2017). Functional organization of cytoplasmic inclusion bodies in cells infected by respiratory syncytial virus. Nat. Commun..

